# Current Management of Amblyopia with New Technologies for Binocular Treatment

**DOI:** 10.3390/vision5020031

**Published:** 2021-06-10

**Authors:** Sandra Boniquet-Sanchez, Noelia Sabater-Cruz

**Affiliations:** Anterior Segment Department, Institut Clinic d’Oftalmologia, Hospital Clinic of Barcelona, 08036 Barcelona, Spain; noelia.sabater@gmail.com

**Keywords:** amblyopia, binocular treatment, dichoptic movies, video games

## Abstract

Amblyopia is the most common cause of monocular poor vision affecting up to 3.7% of the global population. Classically, the first step in treatment has been optical correction, followed by patching and/or pharmacological treatment. However, this is an evolving scenario, since researchers and clinicians are interested in new binocular treatments due to the increasing development of new technologies. In this article main, current binocular treatments as Dig Rush, falling blocks, I-BiT, Occlu-tab, Vivid Vision, and movies are reviewed for binocular amblyopia management.

## 1. Introduction

Amblyopia of the eye, also called “lazy eye”, is a disorder of sight defined as a decreased best corrected visual acuity (BCVA) of one or both eyes without any organic abnormality or pathology of the globe. Clinically, monocular amblyopia is defined as a BCVA of two or more lines less than the fellow eye [1].

Amblyopia comprises a limited visual function including a compromised form, color, and motion perception [2]. In a recent systematic review and meta-analysis published in 2018, Hashemi et al. [3] concluded that the pooled prevalence estimate of amblyopia was 1.8%, with the highest estimate in European Regional Office (3.7%) and the lowest in Africa Regional Office (0.5%).

## 2. Suppression and Cortical Plasticity

Two brain images—coming from dissimilar retinal images on corresponding retinal points—can cause confusion and diplopia. In order to avoid this problem, the brain “ignores” those inputs from the weaker eye under binocular viewing conditions—a process called suppression—so it becomes amblyopic. Stronger suppression of the amblyopic eye has been associated with poorer amblyopic eye visual acuity [4]. The type of amblyopia and its severity do not only adversely affect visual acuity but also binocularity, contrast sensitivity and central versus eccentric fixation.

The age at which children are most sensitive to amblyopia is during the first 2 to 3 years of life, and this sensitivity gradually decreases until the 7 years of age, when visual maturation is complete and the retinocortical pathways and visual centers become resistant to abnormal visual input [4,5]. Early detection is essential to improve treatment success.

The aim of this study is to present the main binocular treatments with a brief explanation of how they work and expound the most important studies related to them. In addition, the effectiveness of classic treatments is compared with that of modern ones.

## 3. Types of Amblyopia

Generally, amblyopia is caused by strabismus (loss of ocular alignment), anisometropia (loss of focus), visual deprivation (loss of form vision), or reverse amblyopia (due to too aggressive amblyopia therapy) [6,7]. A mixed amblyopia is common since strabismus and anisometropia can take place together.

### 3.1. Anisometropic Amblyopia

Anisometropia is the condition in which both eyes have unequal refractive power. Generally, a difference in two or more diopters is the accepted threshold to label the mentioned condition [8]. It can be present in myopic, hyperopic, or astigmatic patients. Moreover, is possible to have an isoametropic amblyopia if both eyes have a similar uncorrected high refractive error and both become amblyopic. Severity of the refractive error and the amblyopia are directly related. According to Hashemi et al. [3], the most common cause of amblyopia was anisometropia (61.6%).

### 3.2. Strabismic Amblyopia

Strabismusoccurs when one eye is not properly aligned, so it may turn inwards (esotropia), outwards (exotropia), downwards (hypotropia), or upwards (hypertropia). The strabismus can be present some of the time (intermittent) or all the time (constant). Constant strabismus led to more severe amblyopia than intermittent ones [2].

Strabismic amblyopia is secondary to strabismus. The brain suppresses the image of the deviating eye as a result of a process to avoid double vision developing anomalous retinal correspondence occasionally [9]. Retinal points in the right and left eyes, which receive stimuli from one object in space, have the same visual direction despite a manifest motor deviation. Patients with anomalous retinal correspondence could have certain degree of peripheral fusion and clumsy stereopsis.

### 3.3. Deprivation Amblyopia

Deprivation amblyopia is the most unusual and, typically, the most severe form of amblyopia. It develops when the visual axis is covered. Various causes of stimulus deprivation include eyelid ptosis, cornea opacities, cataracts, and vitreous hemorrhage, among others [5]. An early treatment of the deprivation etiology is essential to avoid or soften this form of amblyopia.

### 3.4. Reverse Amblyopia

Finally, other infrequent cause of amblyopia is reverse amblyopia. It is a special case of iatrogenic deprivation amblyopia following the patching or atropine excess of the dominant eye and becoming amblyopic as a result [10].

## 4. Methods

Studies were identified limited to English language using PubMed with the search strings “treatment amblyopia”, “dichoptic treatment amblyopia”, “binocular treatment amblyopia”, “dichoptic movies amblyopia”, “video game amblyopia”, “Dig Rush amblyopia”, “Falling blocks amblyopia”, “Vivid Vision amblyopia”, “Occlu-pad amblyopia”, “Occlu-tab amblyopia”, “I-BiT amblyopia”, “perceptual learning amblyopia” and “visual acuity transcranial electrical stimulation perceptual learning”. We searched all available literature without limiting it by publication year until December 2020. The search yielded 287 articles in PubMed that referred to the abovementioned perceptual learning and dichoptic treatments for amblyopia. Titles and abstracts were reviewed excluding duplicates or did not fit in our topic review. After reading the full text of the remaining studies, 35 articles were included in our review, prioritizing the most recent studies and the ones with the largest sample size.

This manuscript is the authors’ own original work, not previously published elsewhere. 

## 5. Amblyopia Treatments

The main objective of amblyopia treatment is even visual acuity between both two eyes, although it is not always achievable. Optical correction is usually the first treatment option and for some children, might be all the treatment needed. When that is not enough, different treatments are required. Some of them rely on depriving the healthy or fellow eye to force use of the amblyopic eye, such as patching or pharmacological treatment. In another treatment, perceptual learning patches the fellow eye, while the amblyopic eye views slowly rotating stripes. The treatments mentioned above are those considered as classical treatments. As it will be explained later on, modern amblyopia treatments are dichoptic or binocular treatments. Regarding binocular treatments, different images are showed to each eye to force work together while the patient is playing or viewing a movie.

## 6. Classical Treatments

### 6.1. Optical Correction

Treatment of the refractive errors is probably the first-line management for amblyopia. For many decades, the Pediatric Eye Disease Investigators Group (PEDIG), the Monitored Occlusion Treatment of Amblyopia Study Cooperative Group (MOTAS Cooperative), and other authors claimed that optical treatment is a good first-line therapy for both anisometropic and strabismic amblyopia [11,12,13]. The amblyopic eye improves the BCVA rapidly during the first 15 weeks when a plateau in visual curve is reached, after which BCVA improves only slowly [11].

A meta-analysis based on 29 articles indicated that although refractive adaptation can improve visual acuity in amblyopic eyes, its effect is significantly decreased with older age. Moreover, visual acuity improves as treatment progresses and a better initial acuity is associated with a higher improvement effect [14].

### 6.2. Patching

Patching is usually an effective second-line treatment for amblyopia. This treatment is based on patching the fellow eye for some hours per day depending on the degree of amblyopia. Usually, the patient has been previously motivated to do near tasks as those activities stimulate the visual system. In 2008, the PEDIG group in a randomized clinical trial found that near and distance tasks had similar effects on amblyopia treatment success [15]. Guidelines from the American Academy of Ophthalmologists [16] and the Royal College of Ophthalmologists [17] advised to use a 6 h patching for severe amblyopia and 2 h occlusion for moderate ones.

In 2019, a large retrospective study of real-world outcomes of amblyopia treatment using PEDIG amblyopia protocols in 877 patients treated at a single center was published [18]. The study population achieved outcomes comparable to those demonstrated by the PEDIG studies in terms of treatment hours.

### 6.3. Pharmacological Treatment

Pharmacological treatment is an alternative treatment for amblyopia when the visual acuity is not fully improved by optical correction, patching is not possible, or a low compliance is detected. The most used drugs are topical atropine and oral levodopa-carbidopa. Their mechanism of action acts by blurring the non-amblyopic eye. Levodopa is converted to dopamine; for the retinal mechanism of action and for cortical mechanism, it has been suggested that increased dopamine levels lead to shrinkage in the size of the receptive field and produce a reduction in the size of the suppression scotoma, respectively, improving visual acuity [19].

In 2002, the PEDIG compared the effectiveness of patching and atropine treatments for moderate amblyopia in 419 children younger than 7 years. Improvement was initially faster in the patching group, but after 6 months, visual acuity improvement was similar in both groups [20]. 

Afterwards, the PEDIG evaluated the effect of daily topical atropine prescribed for the dominant eye in 195 children from 3 years old to younger than 7 years of age with moderate amblyopia. A beneficial effect was demonstrated with atropine treatment, and in 55 children who did not respond properly to atropine alone, a plano spectacle lens was prescribed to improve visual acuity [21].

Moreover, Repka et al. [22] compared daily atropine to weekend atropine treatment for 168 children younger than 7 years with moderate amblyopia. In short, daily and weekend atropine treatment provided similar visual acuity improvement. 

Seol et al. [23] assessed the efficacy of intermittent atropine penalization (one drop in the dominant eye twice a week) for 4 months in 41 children where the mean age was 5.59 ± 1.52. For all the children included in this study, patch therapy had failed previously.

Regarding levodopa as a treatment, Repka et al. [24] compared the efficacy of levodopa as adjunctive treatment to patching by comparing its effect against with treatment with placebo and patching. They found that the group treated with levodopa and patching improved 0.6 and 0.2 logMAR lines in placebo and patching group, respectively.

Likewise, Sofi et al. [25] compare the efficacy of levodopa versus placebo plus occlusion therapy in 50 patients between 5 and 20 years old. As a conclusion, visual acuity improved in both groups but more in the group treated with levodopa. For that reason, they concluded that levodopa-carbidopa can be used as an adjunct to conventional occlusion therapy in amblyopia, particularly, in older children and in severe cases of amblyopia, and it is well tolerated. 

### 6.4. Perceptual Learning

Perceptual learning is another treatment for children and adults with amblyopia. Patients are often trained on contrast sensitivity tasks with occlusion of the non-amblyopic eye. Perceptual tasks are conducted with the Cambridge Visual Stimulator (CAM), a technology from the 1970s using a rotating disc on which high-contrast sine-wave gratings of six different spatial frequencies are displayed. The first grating used is the finest on which the patient can distinguish the orientation of the stripes. After some training, the grating frequencies are being increased, while the visual acuity is improved [26,27]. 

The use of perceptual learning was fairly limited, some studies such as Willshaw et al. [28], in 1980, showed a visual acuity improvement in children with no previous amblyopia treatment and also in those whose previous occlusion therapy had failed. After 4 weeks of completion of treatment, 47 patients were followed-up and 18 of them failed to maintain their visual acuity improvement. This fact brings to light the lack of sustained effects and long-term follow-up using CAM as a treatment. On the other hand, other authors as Tytla and Labow-Daily in 1981 compared a control group undergoing the same procedure without the grating stimulation and a group of patients who followed the CAM treatment [29]. They concluded that the improvements were not significantly different and the vision changes could be attributed to the short-term occlusion.

Recently, an adaptative optics perceptual learning (AOPL) system has been used to measure and correct the high order aberrations in amblyopic eyes [30]. According to a study carried out by Liao et al. [31], adaptative correction may improve the optical qualities of amblyopic eyes.

In addition, two different types of perceptual learning have shown to be effective in clinical research: Gabor’s patches and letter optotypes by training monocularly [32]:. Gabor’s patches are based on the spatial frequency and orientation of a sinusoidal gratings with a Gaussian envelope where orientation discrimination is worked [33,34]. Moreover, letter optotypes are based on the fact that letter recognition is affected by crowding and interaction contours in amblyopia, thus training with letter recognition with and without crowding and orientation discrimination is carried out [35].

Recently, a neuro-modulatory technique, high-frequency transcranial random noise stimulation (hf-tRNS), had been developed and combined with perceptual learning. In a pilot study of Campana et al. [36] and a study of Moret et al. [37], participants underwent 8 training sessions during a period of 2 weeks combining perceptual learning and hf-tRNS showing a VA improvement in patients with amblyopia; in the pilot study, the mean VA improvement was 0.18 logMAR [37], while in the latest study, the mean VA improvement was 0.19 logMAR in the hf-tRNS group [37]. These results are quite positive compared with the results obtained by Polat et al. [34] using only Gabor’s patches for more weeks.

The boredom associated with repetition of a perceptual task over many hours, the dedicated time required for treatment, and need of training systems have limited the use of perceptual learning. An important drawback of this treatment is that the improvements are specific to the trained task and do not transfer easily to novel tasks. Duration of training depends on the severity of the amblyopia without a rule about the specific treatment time required [38]. Another limitation of perceptual learning is the small number of participants in the studies carried out and the lack of sustained effects and long-term follow-up [28]. Consequently, technological improvements have developed new binocular treatments with video games and movies.

## 7. Modern Treatments

The interest and the technological improvements had been developing different treatments for amblyopic patients. Use of interactive devices have been a topic of interest among researchers and clinicians. The aim of binocular treatment is to not only improve visual acuity of the amblyopic eye but also restore binocular fusion and stereopsis. Dichoptic treatment is based on the fact that visual tasks can only be solved if both eyes are working together [39]. 

Currently, there are different ways to improve the visual acuity of the amblyopic eye using videogames or movies. Some of them use red-green anaglyphic glasses, another shutter glasses, others polarized glasses, and another a low-pass filtering that decrease luminance in the fellow eye. The most known binocular treatments are developed below.

### 7.1. Dig Rush

Dig Rush, an action-oriented adventure game, is played on iPad^®^ (Apple^®^ Inc. Cupertino, CA, USA) with a touch-sensitive screen while the patient wears red-green anaglyphic glasses. The game consists in miners digging for gold. The patient has to use a finger to manipulate both miners and their surroundings to dig for gold and return it to a cart as quick as possible while avoiding obstacles such as fire, lava, and monsters [40]. There are 42 levels increasing in difficulty and the patient can earn up to three stars per level (maximum star count, 126). Gold can be used to purchase more miners and digging tools, as well as to dig faster and carry more gold.

**Figure 1:**
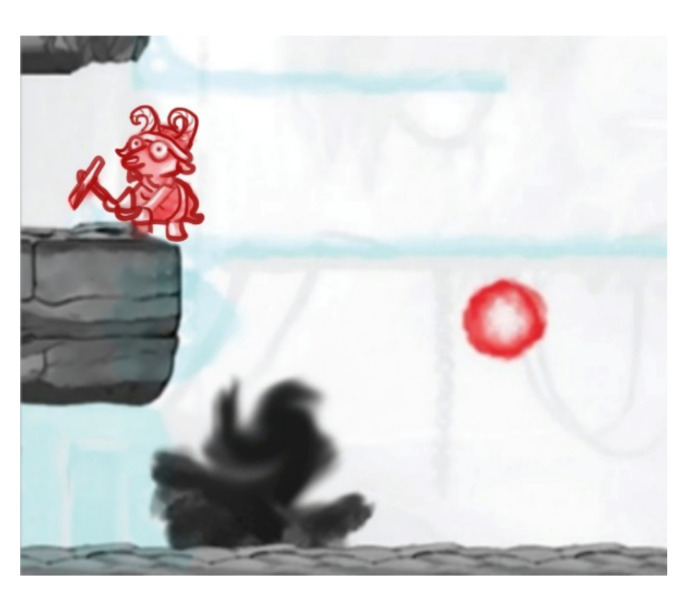
Illustration of Dig Rush. High-contrast red elements (miners and fireball) are seen by the amblyopic eye. Low-contrast blue elements (gold and platforms) are seen by the fellow eye. Gray elements (rocks and ground) are seen by both eyes.

The game is played while the patient is wearing red-green anaglyphic glasses that separate game elements seen by each eye. Reduced contrast elements (e.g., gold and fire) are seen by the fellow eye, high-contrast elements (e.g., miners and monsters) are seen by the amblyopic eye, and high-contrast background elements (e.g., ground and rocks) are seen by both eyes (Figure 1).

In order to succeed playing the game, both eyes must see their respective game components. Amblyopic eye contrast remained at 100% contrast, while fellow eye contrast started at 20% but increased with game success (a star earned) or decreased if game play was not successful. This makes the amblyopic eye works hard together with the fellow eye.

At least 18 h of game play were required to reach 100% contrast to the fellow eye. If game play was unsuccessful for 30 min (no star earned), fellow eye contrast was reduced. The iPad^®^ automatically recorded the duration of game play and contrast to the fellow eye.

Kelly et al. [40] performed a study with 28 children between 4.6 and 9.5 years old with amblyopia due to strabismus, anisometropia, or both. They evaluated the effectiveness of this binocular iPad game as amblyopia treatment and compared this binocular treatment with patching. After 2 weeks, the BCVA improved to 0.15 logMAR lines in the binocular game group versus 0.07 logMAR lines in the patching group. As a conclusion, they affirmed that Dig Rush was effective in treating childhood amblyopia and was more efficacious than patching at the 2-week visit. Treatment duration was really short, it would be recommended to perform it during more weeks.

A few years later, Kelly et al. [41] carried out another study with a biggest sample of 41 children between 4 and 10 years old with amblyopia due to strabismus, anisometropia, or both. After 2 weeks of binocular treatment (20 games and 21 movies), mean amblyopic eye BCVA improved 0.14 logMAR lines from baseline (improvement ranges from 0.0 to 0.4). Besides, they found no difference for change in BCVA between the game and movies treatment. They claimed that binocular treatments that rebalance contrast to overcome suppression are a promising additional option for treating amblyopia.

However, Pediatric Eye Disease Investigator Group and colleagues [42] obtained different results. They evaluated the visual acuity improvement in 138 amblyopic children (resulting from strabismus, anisometropia, or both) aged 7 to 12 years old after 4 or 8 weeks of treatment with the dichoptic binocular Dig Rush iPad game plus continued spectacle correction versus continued spectacle correction alone. All participants had had a prior amblyopia treatment with no improvement in amblyopic-eye visual acuity for at least 8 weeks prior to enrolment.

In this study, adherence data from the iPad was poorer, indicating that slightly more than half of the participants (up to 58%) completed >75% of prescribed treatment by the 4- and 8-week visits. In conclusion, at 4 weeks they found that BCVA improved more in the control group (1.7 logMAR lines) than in the binocular group (1.3 logMAR lines from baseline). In addition, at 8 weeks, the binocular group improved 2.3 logMAR lines and the control group 2.4 logMAR lines. 

In conclusion, Dig Rush did not show more improvement than spectacle correction alone in the PEDIG study mentioned before, but treatment adherence was not so high and it would be interesting to perform more studies with higher adherence. In the other studies with less patients and shorter duration, the improvement had been, approximately, 1.5 logMAR lines. 

### 7.2. Falling Blocks

This is one of the renowned games for binocular treatment. The game was played using an iPod Touch device at the patient’s habitual reading distance while wearing red-green anaglyphic glasses over an appropriate refractive correction. When the amblyopic eye looked through the green filter, only green elements were seen by this eye. In the same way, the fellow eye could see red elements when looked through the red filter. In addition, there were brown elements, which were seen by both eyes. Game elements were presented at 100% to the amblyopic eye and at lower contrast to the fellow eye. Binocular combination was required to successfully play the active video game. Thereby, contrast in the fellow eye was increased, as long as the performance was successful [43].

**Figure 2:**
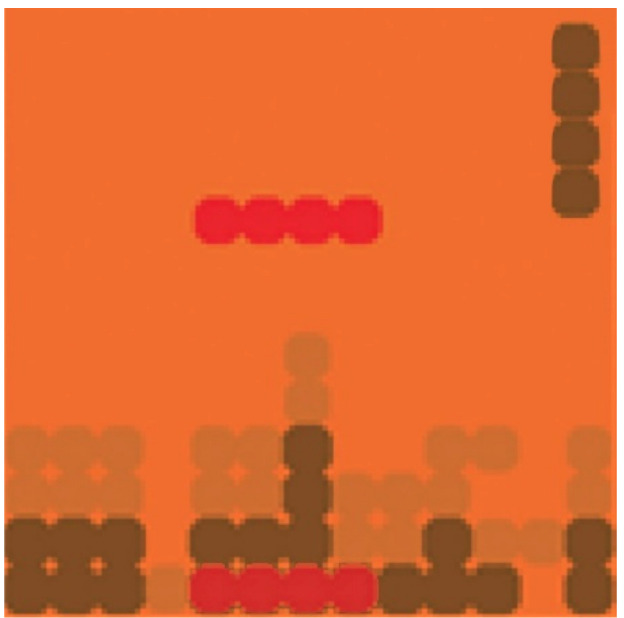
Illustration of falling blocks. Red blocks are seen only with the amblyopic eye, green blocks are seen only with the fellow eye, and brown blocks are visible to both.

Before starting each game session, the participants were asked to align a red-green nonius cross on the device screen in order to compensate small angles of ocular misalignment and assure binocularity. In this game, players had to rotate and move laterally blocks falling from the top of the screen to fit them into gaps left by previously placed blocks at the bottom of the screen. Falling blocks were displayed to the amblyopic eye at full contrast, and placed blocks were displayed to the fellow eye at reduced contrast. Some blocks and a boarder were displayed to both eyes to aid fusion (Figure 2). Participants scored points for each complete line of blocks, and there were different levels of difficulty. The levels differed in the complexity of the block shapes, and whether or not blocks were needed to be rotated during game play.

Gao et al. [44] performed a study with 115 participants with unilateral amblyopia (due to strabismus, anisometropia, or both) aged 7 years or older. They divided the participants by age group: 7–12, 13–17, and 18 years and older. Then, the participants were randomized to receive 6 weeks of active or placebo home-based binocular treatment. The placebo video game presented identical images to both eyes. Falling blocks video game was played for 1 h per day during 6 weeks. At the end, they found an improvement in amblyopic BCVA of 0.06 logMAR lines in the active group and 0.07 logMAR lines in the placebo group. They did not find any statistically or clinically significant differences between groups. Furthermore, separate analyses for the child and adult age groups also did not reveal significant differences between the active and placebo video games.

Previously, in a smaller group of 28 children, between 5 and 14 years old, was evaluated (18 with deprivation amblyopia, 8 with anisometropic amblyopia, and 2 with mixed anisometropic and strabismic amblyopia) [45]. Visual acuity was assessed after a 6 weeks period using the falling blocks game 1 h per day. They found a visual acuity improvement in the anisometropic/strabismic group (0.15 logMAR) and a smaller improvement in the deprivation amblyopic group (0.09 logMAR).

Additionally, the results of this binocular treatment have been compared with patching. Manh et al. [46] enrolled 100 participants aged 13–17 years with amblyopia resulting from strabismus, anisometropia, or both. Participants were randomly assigned to treatment for 16 weeks of either a binocular iPad game prescribed for 1 h per day (*n* = 40) or patching of the fellow eye prescribed for 2 h per day (*n* = 60). After 16 weeks, the BCVA in the binocular group and the patching group improved 0.74 lines and 1.26 lines, respectively. They concluded that patching was more effective than binocular treatment. However, in the binocular group, treatment adherence was low, i.e., data from the iPad device indicated only 13% of participants completed >75% of prescribed treatment.

Apparently, falling blocks has not proven to be an effective treatment so far. Gao et al. [44] almost found the same improvement in the placebo group than in the active group. Hamm et al. [45] found an improvement that might be similar to test–retest repeatability —less than 1.5 logMAR lines—and may not be clinically significant for amblyopes [47]. In addition, Manh et al. [46] showed that patching was more effective than treatment with falling blocks. However, treatment adherence in the binocular group was low, and more randomized controlled trials with high adherence could be interesting.

### 7.3. I-BiT

A variant modality of amblyopia therapy is interactive binocular treatment (I-BiT™). I-BiT™, developed by the Nottingham team [48], is based on three different mechanisms: first, by presenting fine and movable stimulus to the amblyopic eye and the fixed targets or background to the dominant eye; second, by showing the half of one image to each eye simultaneously; and third, by demonstrating identical images to both eyes with small retinal disparity. Binocular fusion is guaranteed thanks to the simultaneous stimulus presentation to both eyes. This system gives the possibility of adjusting its illumination and image contrast according to the patient’s visual acuity. During the treatment, the participant wore a shutter glasses, which lighten and darken in synchrony with the monitor but faster than the user can perceive. This allows a common background to be presented to both eyes and an “enriched” image to be presented only to the amblyopic eye, i.e., dichoptic stimulation.

As time goes by, more games have been created to attract the child’s cooperation, e.g., a variation on the game Nux. The I-BiT system can also display video footage. Besides, an eye-tracker has been incorporated in the I-BiT system for home use.

In the study by Herbison et al. [48], 75 children between 4 and 8 years old with strabismic, anisometropic, or mixed amblyopia were recruited. These patients were randomized to one of three treatments: I-BiT game, non-I-BiT game, and I-BiT DVD. The I-BiT game group played an interactive game called Nux using the shutter glasses, where obstacles, enemies, and coins are shown only to the amblyopic eye, whereas in the non-I-BiT game version, both eyes receive identical stimulation. Regarding I-BiT DVD group, stimulus is divided into two zones. There is an outer “border” termed a locking stimulus, which is presented to both eyes while the inner part of the screen presents the video footage predominately to the amblyopic eye. Each group received their randomized treatment weekly for 30 min period during 6 weeks (3 h total). There was a modest BCVA improvement in the amblyopic eye at 6 weeks in all three groups: 0.06 logMAR in the I-BiT game, 0.03 logMAR in the non-I-BiT game, and 0.01 logMAR lines in the I-BiT DVD. Hence, there was no difference between I-BiT DVD and non-I-BiT games compared with I-BiT games in terms of gain in vision. A very short treatment duration, only 3 h when compared with patching, can be highlighted as a limitation, for example.

Independently of the Nottingham team, an Iranian group developed their own system, which they also called I-BiT, using dichoptic games (Pacman, Tetris, and Snake) with red-green anaglyphic glasses. In 2016, Rajavi et al. [49] carried out a study with this system. They evaluated the effectiveness of I-BiT accompanied with patching in amblyopia therapy. Fifty unilateral amblyopic children between 3 and 10 years old were randomly classified into the case and control groups (25 in each). The case group received dichoptic treatment and patching, whereas the control groups received only patching. The case group played games for 20 min in each session for 5 days a week within 1 month (total time: 6.6 h) and patching for 1 month more in both groups. Eventually, they found an improvement in BCVA of 0.17 logMAR lines in the children who played with the video games, whereas the control group showed an improvement in BCVA of 0.07 logMAR lines. The biggest limitation of this study was treatment duration.

Three years later, Rajavi et al. [50] improved the previous study comparing the effect of amblyopia therapy on cases who received interactive binocular treatment (I-BiT) with those who received standard patching of the dominant eye with placebo I-BiT. In this randomized clinical trial, 38 unilateral amblyopic children between 3 and 10 years old were studied. Children were randomly divided in case group (those played 20–30 min per day for at least 5 days in a week for 1 month with red-green glasses, 6 h total) and the control group (those who played 20–30 min per a day for at least 5 days in a week for 1 month without red-green glasses and also 2 or 4 h patching of dominant eye per day for mild and moderate amblyopia, respectively). In the end, the team concluded that I-BiT game and patching with placebo game had similar BCVA improvement, 0.07 logMAR lines and 0.09 logMAR lines, respectively, in amblyopic children after 1 month treatment. Even though, the compliance was high (87.5% in cases and 76% in controls), treatment duration was too short. 

To sum it up, studies have shown that using I-BiT for less than 6 h does not seem to influence in the BCVA improvement.

### 7.4. Occlu-Pad or Occlu-Tab

**Figure 3:**
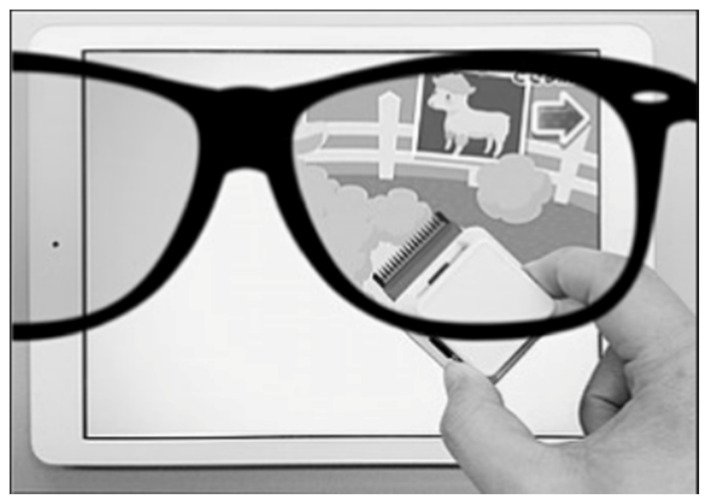
Appearance of the Occlu-pad. Only the right eye can see the image of the tablet terminal, while the left eye cannot.

Occlu-pad is a device that processes images presented selectively to the amblyopic eye under open binoculars. Occlu-pad uses white-screen technology and polarized glasses. The white-screen technology involves peeling off the polarizing film layer of a liquid crystal panel of an iPad, and, by attaching this peeled film to glasses, viewing videos is only possible when the subject is wearing the polarized glasses. For example, if the film is attached to the right-eye lens of glasses, the subject can view the image only in the right eye (Figure 3). During training using the Occlu-pad, the patient played an arbitrary game requiring eye-hand coordination [51]. In Japan, this device is named “Occlu-pad” but outside Japan, it is named “Occlu-tab” [52].

In 2018, Iwata et al. [52] were the first to report the effectiveness of Occlu-pad training for anisometropic amblyopia in a series of cases without patching. They enrolled 22 children (4.7 ± 1.2 years) with anisometropic amblyopia and assessed the visual acuity at 3 and 6 months after training with Occlu-pad. As a result, they found an improvement in BCVA of 0.19 logMAR lines on an average after 3 months and 0.29 logMAR lines after 6 months, being the compliance rates 88.6% ± 18.9% and 73.2% ± 18.9%, respectively.

Afterward, Iwata and the same colleagues [53] recruited more patients and investigated amblyopia training effects using Occlu-pad in a hospital setting in 46 children between 3 and 7 years old. All patients were diagnosed with anisometropic amblyopia and wore full correction in glasses. Twenty-three of the children were treated for amblyopia with only glasses “Glasses treatment group”, while the other 23 patients were treated with glasses in combination with the Occlu-pad “Occlu-pad treatment group”. The last group played an arbitrary game requiring eye-hand coordination with the Occlu-pad for 2 days a week (30 min per day) for 6 months. The compliance rate for using the Occlu-pad was 88.4 ± 18.7% after 3 months and 69.6 ± 19.5% after 6 months. They concluded that although BCVA significantly improved in both groups, more improvement in the Occlu-pad treatment group was observed. The glasses treatment group improved 0.1 logMAR lines at 3 months and 0.19 lines at 6 months, whereas the Occlu-pad treatment group improved 0.19 logMAR lines at 3 months and 0.30 logMAR lines at 6 months.

The same year, Totsuka et al. [54] examined visual acuity improvement effect and adherence in amblyopia training using Occlu-pad versus patching. The subjects were 138 children between 3 and 9 years old with amblyopia due to anisometropia or strabismus. Seventy-two underwent Occlu-pad training and 66 underwent patching training. Visual acuity improvement was evaluated at 3, 6, 9, and 12 months and was compared with baseline BCVA (at the start of training). After the treatment, both groups showed improvement. For anisometropic amblyopia, the Occlu-pad group and the patching group showed an improvement in BCVA of 0.69 and 0.47 logMAR lines, respectively, after 6 months. In addition, for strabismic amblyopia, the BCVA in the Occlu-pad group and the patching group showed an improvement of 0.59 and 0.38 logMAR lines, respectively, after 9 months. Regarding training adherence, the Occlu-pad group showed significantly higher adherence than the patching group after 3 months of training and also throughout the study.

Although there are not a great number of studies related to Occlu-pad, for the time being, amblyopia training with Occlu-pad supports greater visual acuity improvement than only optical correction. However, this improvement was not statistically significant, it was less than 1.5 logMAR lines. On the other hand, in other studies, this binocular treatment had shown a greater visual acuity improvement—around 2 logMAR lines—and better adherence than patching.

### 7.5. Vivid Vision

**Figure 4:**
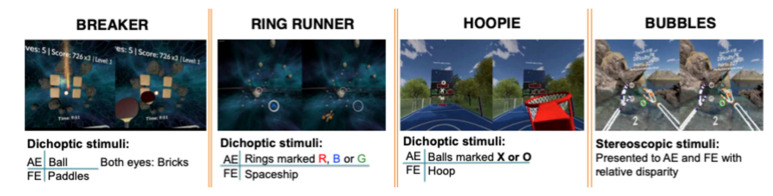
Illustration of four games provided by Vivid Vision.

Last but not least, mention the computer game called Diplopia Game (Vivid Vision, San Francisco, CA, USA), which was run in the Oculus Rift OC DK2 virtual reality head mounted display (Oculus VR) [55]. At first, two games were available, i.e., a space game, in which subjects were flying spaceship through a system of rings, and a breaker game, which is a typical block breaker game, but played in a virtual reality 3D setting. Next, they incorporated Hoopie, a game where the aim is to catch or refuse some objects using the hoop, and Bubbles, where the objective of the game is pop the floating bubbles in order from closest to farthest away. As some objects are seen with the amblyopic eye and others are seen with the fellow eye, the game forces the brain to use both eyes together (Figure 4). 

There is not a great number of studies published with this kind of device, but those ones that are published showed good results. Ziak et al. [56] evaluated 17 adults between 17 and 69 years old with anisometropic amblyopia. All the participants performed 8 training sessions, twice a week and 40 min long each session (20 min playing Breaker game and 20 min playing Ring runner game). After treatment, an improvement in BCVA of 1.5 logMAR lines on average was found in most of the patients, except in four cases where no change in BCVA was observed. The main limitation of this study was the small number of participants.

Ho et al. presented an abstract at the 2019 ARVO Annual Meeting [57], confirming the favorable outcomes of Vivid Vision. The objective of this independent assessment was to define the clinical efficacy of this dichoptic training in the treatment of amblyopia using low-pass filtering (“blur”) and decreased luminance (“occlusion”) in the fellow eye. Thirty-four patients between 3 and 69 years old with amblyopia due to anisometropia, strabismus, or both were enrolled. Thirty-one of them played for 8 weeks (30 min per session each week) and 3 participants only 4 weeks. Finally, they found an improvement greater than 1.5 logMAR lines in the BCVA in the amblyopic eye and they saw a stereoacuity improvement (around 100 s of arc) in participants who had measurable stereopsis at the start of treatment (*n* = 15).

Additional research with randomized controlled trials is required in order to evaluate the usefulness of virtual reality training for the improvement in visual acuity in amblyopia. For the time being, the few studies carried out with Vivid Vision had led improvements equal or greater than 1.5 logMAR lines.

### 7.6. Movies

It is clear than not all the patients know how or want to play video games, and moreover, some patients are so amblyopic that it is not possible to play video games. Passive dichoptic movies are a good alternative in those cases.

A digital mask composed of irregularly shaped blobs is applied on the images seen by the amblyopic eye, and an inverse mask was applied to the images seen by the fellow eye. So, the procedure is based on the presentation of complementary images in the two eyes while the patient wears polarized glasses. Thus, to perceive a completed coherent picture, it is necessary to combine information seen by both eyes. The shape and location of the blobs vary dynamically every 10 s. The contrast of the image seen by the amblyopic eye is fixed to its maximum, and the contrast of the image seen by the fellow eye is based on the results of the binocular balance contrast sensitivity baseline measure. If the subject perceives the full picture of the movie during a session, the contrast in the fellow eye was increased for the next session.

**Figure 5:**
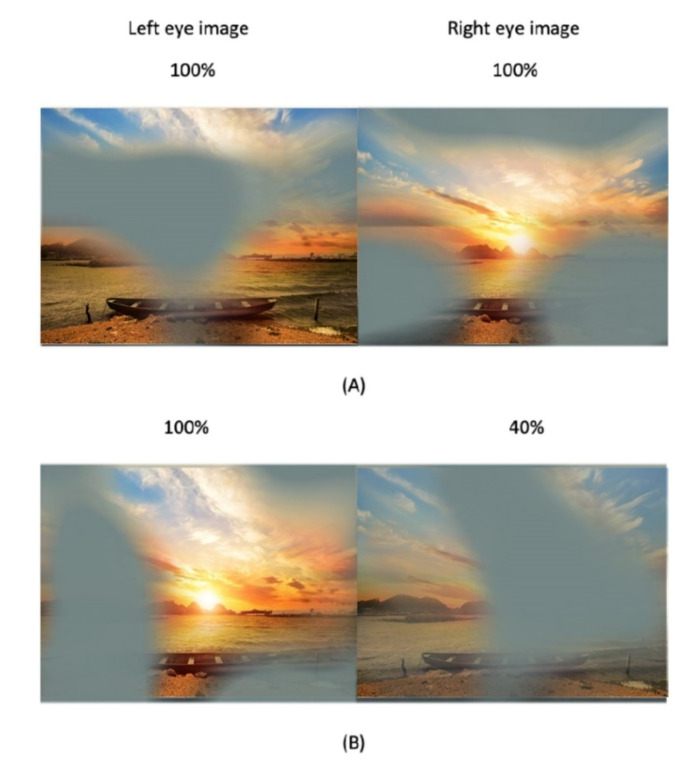
Illustration of the dichoptic movies. The two eyes’ views are shown side by side. Complementary patterned image masks composed of irregularly shaped blobs were overlaid over the images seen by the two eyes. The shape and location of the blobs were varied dynamically every 10 s. (**A**) 100% contrast images were presented to the two eyes. (**B**) A 100% contrast image is presented to the left eye, and an image with a contrast reduced to 40% is presented to the right eye.

Two recent studies examined the effect of binocular amblyopia treatment with contrast-rebalanced movies in different patients. Birch et al. [58] assessed 27 amblyopic children (due to anisometropia, strabismus, or both) between 4 and 10 years old after watching 6 contrast-rebalanced dichoptic movies on a passive 3D display during a 2-week period. Amblyopic eye contrast was 100% and fellow eye contrast was initially set to a lower level (20–60%), which allowed the child to overcome suppression and use binocular vision (Figure 5). Fellow eye contrast was incremented by 10% for each subsequent movie. As a result, amblyopic BCVA improved from 0.57 logMAR at baseline to 0.42 (improving 0.11 logMAR). Children aged 3–6 years had more improvement (0.21 logMAR) than children aged 7–10 years (0.11). Also, they found more improvement in children with severe amblyopia than children with moderate amblyopia at baseline, with 0.24 and 0.12 logMAR improvement, respectively. Although the sample size was small and treatment duration was short, their results suggested that passive viewing of dichoptic animated feature films is a feasible and effective amblyopia treatment.

On the other hand, Sauvan et al. [59] evaluated, in 17 patients, the effect of treatment with dichoptic movies versus dichoptic movies after 2 h wearing an occluding patch in old children and adults (between 9 and 67) with stable and resistant amblyopia. In other words, patients with stable visual acuity for at least 1 year before inclusion and children under 12 years old had to go through at least 6 months of conventional occlusion therapy to make sure that amblyopia was stable and resistant. Finally, their results showed that even very short dichoptic movie viewing significantly improved BCVA from 0.54 to 0.46 logMAR in the amblyopic eye in the non-patched group and from 0.62 to 0.43 logMAR in the patched group after, approximately, 9 h of training over a 2-week period. The small sample size of the study was another great limitation. As a result, the addition of a short-term monocular occlusion to the dichoptic training shows promising trends but was not significant for the sample size used there. They affirmed that passive movie approach combined with interocular contrast balancing even over such a short period as 2 weeks had potential as a clinical therapy to treat amblyopia in older children and adults. 

Furthermore, it stands out that in the study carried out by Kelly et al. [41], 21 of the children were treated with passive movies (approximately, 9 h of treatment during 2-week treatment period) that used the same system of the shaped blobs.

**Figure 6:**
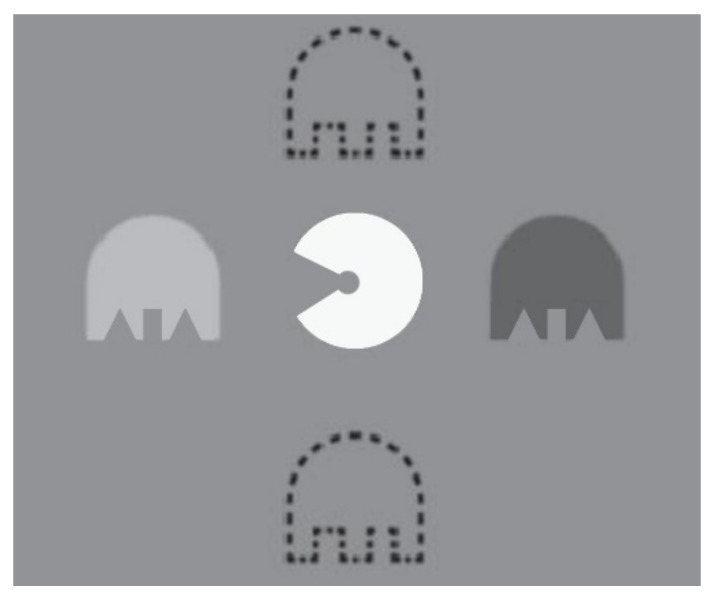
Illustration of VacMan flanked by two ghosts either positioned on the left and the right (as shown) or above and below (dashed outlines). Each ghost was a mixture of one dark and one light ghost presented to different eyes on each side (illustrated within the white circles). Suppression is quantified as the mixture of luminance required for the child to be equally likely to report either ghost as “whiter”.

Alternatively, Bossi et al. [60] used another type of dichoptic movie treatment. They presented three-dimensional (3D) movies and used shutter glasses to control the image presented to the two eyes. The image received by the fellow eye is blurred up to equate the amblyopic eye visual acuity. During treatment, the movie was interrupted every minute by an interactive game used to measure suppression. Two dichoptically presented “ghosts” flanked a central VacMan, either above/below or left/right (Figure 6). Children were provided with a keypad and they have to answer where the whitest ghost is. In this study, 22 children between 3 and 11 years old with amblyopia (due to anisometropia, strabismus, or both) spent on average 75 h and 14 min on treatment. Before treatment, mean BCVA was 0.78 logMAR and after treatment, it was 0.51 logMAR, i.e., improvement of 0.27 logMAR.

To sum it up, all the studies mentioned above showed a visual acuity improvement to a greater or a lesser degree. Birch et al. [58] found an improvement of 0.1–0.2 logMAR in 81% of the patients, 0.3–0.4 logMAR in 14% of the patients, and only one child did not show any improvement. Sauvan et al. [59] found more improvement in the patched group (0.19 logMAR) than in the non-patched group (0.08 logMAR). In addition, Bossi et al. [60] also described a mean improvement in acuity of 0.27 logMAR for the amblyopic eye. 

## 8. Discussion

Nowadays, a few studies have compared classical with modern treatments in amblyopia [40,42,44,46,48,49,50,53,54,59]. Some of them compare the effect of patching versus binocular treatments [40,46,49,54,59]. Others compare binocular treatments with placebo video games [44,48,50], and two studies compared sole optical correction versus binocular treatments for amblyopia management [42,53]. 

Results of studies comparing binocular treatments versus patching are diverse: Binocular treatment such as Dig Rush or Occlu-pad resulted to be more effective than patching [40,54]. I-Bit accompanied with patching improved BCVA more than only patching [49]. On the other hand, patching showed a major BCVA improvement than falling blocks [46]. As for movies, treatment with dichoptic movies versus dichoptic movies after patching 2 h, a greater BCVA improvement was found in the patching group [59]. 

Those studies that compared binocular treatments with placebo video games—as falling blocks and I-BiT—did not show significant differences in BCVA improvement [44,48]. In addition, the effect of I-BiT game and patching with placebo game showed similar BCVA improvement after 1-month treatment [50].

When binocular treatment is compared with sole optical correction, the results are also assorted. Holmes et al. [42] compared both treatments in children aged 7 to 12 with previous treatments for amblyopia other than spectacles, concluding that there was no benefit to visual acuity from 4 to 8 weeks of treatment with the dichoptic binocular Dig Rush iPad game. In contrast, Iwata et al. [52] compared the effects of Occlu-pad treatment with spectacle correction alone in children between 3 and 7 years old, noting that the BCVA improvement in the Occlu-pad group was greater even though they were not statistically significant.

Regarding the potential factors that could influence the variability of findings across different studies is important to highlight the following aspects. Some studies have a small sample size, reducing in this way the power of the study and increasing the margin of error [53,56,59,60]. Moreover, in some studies, treatment duration was short, 2 weeks or less than 9 h [40,41,48,49,50,58,59]. Other potential factor that might influence the results was the low treatment adherence [42,46]. Lastly, mention that not all the studies had a control group [45,53,56,60].

The primary limitation of this review is the literature research. This is not a meta-analysis, however, it is rather a narrative review.

Most of the studies reviewed had not shown a statistically significant improvement in the BCVA in amblyopic patients after binocular treatments. In those studies where the BCVA improvement was less than 1.5 logMAR lines, it could be considered within the test–retest repeatability levels for amblyopes [41,42,44,45,46,48,50,59]. Nevertheless, other studies reviewed before resulted to improve the BCVA greater than or equal to 1.5 logMAR lines [40,42,45,49,52,53,54,56,57,58,60].

Studies had linked the level of compliance and treatment hours to favorable results in amblyopia management. With binocular treatments based on games and movies, the adherence rate is improved better. Nonetheless, treatment duration—in terms of number of sessions—is essential: if too short, the desired effect may not appear, but if too long, the compliance could decrease because of boredom or lack of interest.

## 9. Conclusions

In summary, technological development in the last decades has been explosive, leading to an innovative and improved management of the amblyopia based on binocular treatments. Future investigations with randomized controlled trials are needed to better understand and improve study results for binocular amblyopia.

## Data Availability

Not applicable.
